# Effect of gravity on the spreading of a droplet deposited by liquid needle deposition technique

**DOI:** 10.1038/s41526-023-00283-2

**Published:** 2023-06-21

**Authors:** Aleksey Baldygin, Abrar Ahmed, Ryan Baily, Md Farhad Ismail, Muhammed Khan, Nigel Rodrigues, Ali-Reza Salehi, Megnath Ramesh, Sanjay Bhattacharya, Thomas Willers, Derek Gowanlock, Prashant R. Waghmare

**Affiliations:** 1 interfacial Science and Surface Engineering Lab (iSSELab), Department of Mechanical Engineering, University of Alberta, Edmonton, AB T6G2G8 Canada; 2 Engineering Beyond INC., Edmonton, AB T5J4P6 Canada; 3 Krüss GmbH, Borsteler Chausse 85, Hamburg, Germany; 4 Aerospace Research Centre, National Research Council Canada, 1920 Research Rd, Bldg U-61, Ottawa, ON K1V2B1 Canada

**Keywords:** Fluid dynamics, Mechanical engineering

## Abstract

This study represents an experimental investigation, complemented with a mathematical model, to decipher the effect of gravity on the spreading dynamics of a water droplet. For the theoretical discussion, an overall energy balance approach is adopted to explain the droplet spreading under both microgravity (*μg*) and terrestrial gravity condition. Besides explaining the mechanism of the droplet spreading under microgravity condition achieved during the parabolic flight, a technique with a detailed experimental set-up has also been developed for the successful deposition of droplet. A rational understanding is formulated through experimental investigation and theoretical analysis, which allows us to distinguish the transient variation of the spreading of a droplet, between microgravity and terrestrial gravity condition. The spreading of the droplet is predicted by the non-linear overall energy balance equation, which accounts for the operating parameters in the form of non-dimensional groups like Reynolds number (${{{\rm{Re}}}}$), Weber number (We) and Bond number (Bo). To distinctly identify the difference in the drop spreading at terrestrial and microgravity conditions, the Bo with transient gravitational field obtained through the on-board accelerometer is considered. The obtained theoretical results are further corroborated by experimental results which are obtained from the parabolic flight.

## Introduction

The fundamentals of capillarity and spreading phenomenon are dictated by the interplay between the interfacial and body forces like surface tension and gravitational force. In the absence of gravity this interfacial force dominates the most of the liquid behaviours. Therefore thorough painstaking research has been conducted to understand the influence of gravity on the interfacial phenomenon. In this endeavour, a considerable amount of literature have been devoted to the effect of gravity on capillary-driven phenomena. Referring to the classical theory of capillarity^1^, if the characteristic length of a drop is less than a capillary length, gravitational effects can be neglected and hydrostatic pressure rapidly stabilizes across the droplet profile. It leads to a spherical shape being adopted by the droplet in order to obey the Laplace law.

Droplet spreading or wetting is one of the ubiquitous phenomena that is governed by interplay between interfacial forces and has a wide range of industrial applications. In nature, several intriguing phenomena are dictated by the wetting such as the self-cleaning property of lotus leaves^2^, the water strider walking on water surfaces^3^, the anti-fogging functionality of mosquito eyes^4^, the water collection of the Namib Desert beetle^5^, and so on^6–8^. On the other hand, the knowledge of spreading dynamics is a fundamental of many industrial-based applications, including but not limited to inkjet printing^9^, bio-sensors^10^, spray coating^11^, agriculture^12^, 3D printing^13^ and many more. Therefore, the understanding of the physics of droplet spreading is crucial for the development of nature-inspired, state-of-art research.

Theoretical explanation of the gravitational effect on the contact angle of a droplet has already been presented by many researchers^14–16^. Fujii et al.^17^ developed a drop shape model where the curvature of the drop was a function of gravity. Herzberg and Marian^18^ have experimentally investigated that the change in the contact angle does not depend on the drop size, rather it is primarily due to the change in the contact angle hysteresis, however, they did not test their hypothesis on reduced gravity environment. Later on, Good and Koo^19^ attempted introducing a hypothetical negative line tension to justify the effect of droplet size on the contact angle variation. Performing meticulous mathematical exercise and rigorous calculation based on Bashforth and Adams^20^ scheme, Fuji and Nakae^17^ showed that the equilibrium contact angle is unaffected by the gravity.

However, the clarity is still missing, whether the physical and interfacial properties of fluid are affected by the gravitational force or not? Due to the higher expense and accessibility to reduced gravity environment at the International Space Station (ISS), researchers have attempted to simulate the reduce gravity environment instead of going to space with fairly accessible parabolic flights^21^ or drop tower facilities^22^. In the case of droplet dynamics, with either ways, unfortunately, drop deposition always remained the biggest engineering challenge, in particular if the drop deposition is achieved during the reduced gravity time span. This time span is a few seconds (2-2.5 seconds) for drop tower and between 15 and 25 s for parabolic flights. Hence, significant efforts have been devoted to engineer drop deposition technique that unaltered the wetting or spreading of the droplet. In the current study, presented by us, we proposed a technique, which circumvents most of the undesirable effects associated with the deposition technique. After the flight campaign, we are convinced that this can be the next-generation drop deposition techniques for reduced gravity applications and we have vetted it for the wetting characteristic and drop spreading dynamics applications, where the drop deposition is the key step.

With terrestrial conditions, drop deposition can be achieved using techniques such as drop deposition with the needle facing the substrate^23^, drilling substrate^24,25^ and other specific needle-less drop deposition techniques^26–29^. So far, for the reduced gravity applications, majority of studies have drilled the substrate to pump liquid from underneath the substrate, which eventually forms the drop on the substrate^30^. Alternatively, drop is deposited on a surface prior experiencing the reduced gravity and deformation in equilibrated drop shape is studied^31^. In both the circumstances, experimental arrangements were restricted from studying the spreading dynamics, thus instantaneous spreading in reduced gravity environment has not been studied yet. Ababneh et al.^30^ experimentally investigated, using parabolic flight, the effect of gravity on the advancing contact angle after depositing the drop before the drop experiences the reduced gravity. In their work, advancing contact angle in the terrestrial gravity is reported 5^∘^ larger than that in reduced gravity. Later on, Zhu et al.^32^ experimentally investigated the contact angle dependence of an evaporating sessile and pendant drop on the microgravity. However, they have observed that the equilibrium or apparent contact angle of a water droplet on aluminium substrate is decreased by 15^∘^ in microgravity^32^. Diana et al.^31^ initiated the development of a database of contact angles of sessile droplet under reduced gravity conditions. Based on the database presented by Diana et al.^31^, two observations can be made, in all of the studies the drop is deposited before the reduced gravity triggers and the measured contact angles are always smaller in magnitude as compared to the terrestrial measurements. From this study it is also evident that the Young-Laplace equation was validated to accurately predict the contact angle in reduced gravity for droplets smaller than capillary length scale; however, it was not adequate to describe the contact angle for drops larger than capillary length scale. One limitation of this study is the duration of the reduced gravity drop can experience, and is limited to 2.2 s. Brutin et al.^33^ have witnessed two different contact angles depending on the onset of water droplet generation. If the drop is already equilibrated before it goes the microgravity, the contact angle can be 10^∘^ lower compared to the same drop created under microgravity condition^33^.

Despite a fair number of publications have been devoted to the experimental investigation on the variation of physical parameters of a droplet with respect to gravity, a fundamental model describing the droplet spreading phenomenon under reduced gravity condition is still missing from the literature. Additionally, a reliable and reproducible drop deposition technique under reduce gravity condition is yet to be addressed. Thus, our present study addresses a droplet deposition technique functioning under microgravity and proposes a mathematical model, which can predict the spreading of the three-phase contact line diameter of a droplet both under reduced and terrestrial gravity condition. The theoretical model presented here is based on an overall energy balance equation, where dimensionless numbers, such as the Reynolds number, the Weber number, and the Bond number characterize the droplet spreading. Furthermore, the jet impact analysis is introduced in order to define the initial condition while quantifying the transient variations in the geometrical parameters of a droplet. Finally, we compare our theoretical predictions with the experimental results, obtained in parabolic flight, which was a part of flight campaign sponsored by Canadian Space Agency through FAST Grant that took place in October 2021 at Flight Research Laboratory. The experimental set-up was previously verified in parabolic flight, which was part of the inaugural Canadian Reduced Gravity Experiment Design Challenge (CAN-RGX) flight campaign and results reported here are from recently performed flight campaign.

## Results and discussion

### Drop deposition in *μ*g

The conventional drop deposition such as droplet volume method or sessile droplet method have their limitation in reduce gravity experiment, perhaps they will fail in this case^34^. Droplet weight or volume method^35^, where the droplet is detached from the capillary by its own weight, is not a valid choice to deposit droplet on the substrate. The pendant droplet technique, where the drop is brought in the close proximity to the substrate and allowed it to detach from the needle, is also not a viable option as it poses numerous engineering challenges^26^. Moreover, for parabolic flight experiments, the time window to perform experiments is between 18–20 s, as shown in Fig. 5 and the “g-jitter” plays a crucial factor while deciding the drop deposition technique^36^. Similarly, for the drop tower, a proper drop deposition technique has not been achieved that can work during the reduced gravity time (~2.2 s) window. To the best of our knowledge, all the previous literature on reduce gravity experiment in parabolic flight or drop tower facility describe the generation of droplet on the substrate through the quasi-static addition of mass by pumping a liquid through a hole in the substrate, where the position of the needle is underneath the substrate^24,30,31^. However, pumping liquid by a needle through the punctured substrate has major drawbacks, as it is sensitive to the injection mass flow rate and the appropriate ratio between the drop and needle diameter. If the injection mass flow rate is high enough then there will be formation of jet from the needle instead of a droplet^32^. On the other hand, if the injection of mass flow rate is low, it will grow at drop-medium interface rather spreading at the three-phase contact line and the evaporation of liquid can also take place during the slower drop generation^37^. Considering all the adverse effects of existing drop deposition method in microgravity, a jet-based drop deposition method, also known as liquid needle method^29,38,39^, is proposed here. In liquid needle drop deposition technique, a very thin jet (100 μm) of constant flow rate is generated that can pump out the fluid through jetting at constant jet velocity up to 25 μl s^−1^^38,39^. It is proven that, on terrestrial conditions, a reproducible sessile drop volumes with the same equilibrium contact angles can be produced with this deposition technique^38,39^. It is also proven that if the ratio of the diameter of the jet to the diameter of the drop is 0.2 or less, the adverse effect of kinetic energy^38,39^ on the reproducibility of the recently advanced contact angle can be minimized^37^. Therefore, considering all the shortcomings of traditional droplet deposition techniques and other approaches, we have decided to extend the utility of a jet-based drop deposition technique^29,39^ to deposit or generate the droplet onto the substrate under reduce gravity condition.

It is noteworthy to mention that numerous piezoelectric and pneumatic drop on demand (DOD) generators are available in the market. However, all the commercially available DOD generators are developed for pharmaceutical or ink-jet applications where the inherent kinetic energy due to the impacting droplets is significantly higher. To the best of our knowledge none of the existing devices are ideal for the droplet deposition and wetting characteristic due to the inherent kinetic energy with ejecting fluid that contribute towards the erroneous contact angles^29,38,39^. Additionally, commercially available pneumatic DOD devices are more prone to generate satellite droplets which are not desirable in the microgravity environment^40^. Finally, cyclic acceleration (2*g* to ~0*g*) involved in the parabolic flights and limited space inside the flight cabin mandates the robustness, compactness and requirement of less number of components with minimal energy consumption for a component or device.

Using a liquid jet for the purpose of generating a spreading droplet comes with challenges. Usually in order to form a continuous jet, a necessary criteria must be fulfilled such as Rayleigh stability or high Weber number^41–43^. As mentioned earlier, the higher kinetic energy might have a considerable influence on the spreading and resultant contact angle. More precisely, if the liquid jet is impacting on a substrate, with significantly higher speed (i.e., kinetic energy and resultant Weber number), the kinetic energy in the drop translates into the formation of the drop and overspreading of drop might be witnessed or in some cases splashing with formations of multiple droplets. As a result, either drop formation cannot be achieved or the measured contact angles with formed drops is incorrect representation of wettability of the substrate. Conversely, if the jet speed is low enough, the break-up of the liquid jet before hitting the substrate can cause the instabilities at the drop medium interface and triggers the air bubble formations inside the drops, resulting in falsified contact angle results. Therefore, the speed at which the jet emanates from the nozzle needs to be controlled carefully in order to get an impeccable wetting results, which in general is cumbersome with the commercial DOD generator.

**Fig. 1:**
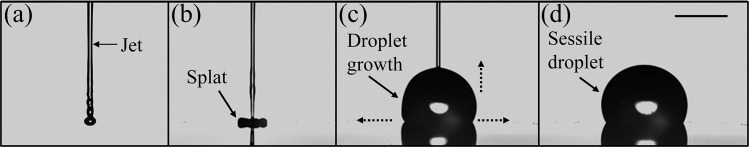
Snapshots representing the formation of a droplet through liquid needle drop deposition technique. **a** The water jet formation in the air medium, (**b**) liquid jet impacting on a substrate and forming a splat, (**c**) droplet growth due to the continuous addition of mass in the form of liquid jet where the horizontal and vertical dotted arrow signs indicate the spreading of base diameter and the increment of droplet height, (**d**) sessile droplet created by the liquid needle drop technique. The scale bar showed in panel (**d**) represents 1 mm.

The liquid needle drop deposition technique^39^ is precisely studied to circumvent these unavoidable effects and optimized in such a way that the influence of the kinetic energy on the measured contact angles is negligible. To achieve this, the flow rate of the water is optimized at 15 ± 2 μl s^−1^, which assures a continuous laminar liquid jet emanating from a pressurized dosing system^29,38^. This laminar jet is used to generate the sessile drop, as shown in Fig. 1, where the droplet size is generally one order of magnitude higher than the jet diameter^29,38^. As shown in Fig. 1, the jet spreads radially outward from the point of contact as soon as the liquid jet hits the surface due to the jet’s kinetic energy. The continuous mass addition through jet results in an increase in the base diameter and height of the spreading droplet. It is noteworthy to mention that, unlike the single droplet impact scenarios, rapid retraction of the spreading drop at the three-phase contact line or other undesirable effects due to resultant capillary waves^39^ are restrained during liquid needle drop deposition technique. Hence, we can say that in the case of liquid needle deposition technique, the simultaneous spreading is achieved along with the mass addition via jetting across the drop-medium interface.

The jet parameters used in this study are tuned optimally for a given liquid-surrounding medium in such a way that the magnitude of the momentum imparted on the drop negligibly impacts the later-stage drop spreading and resultant equilibrium contact angle. The details of the theoretical understanding are described in the Supplementary Discussion of the paper. Droplet spreading is also influenced by the surface energies of the liquid and solid for a given surrounding medium, viscous dissipation within the drop and from the surrounding medium, and gravitational forces. If the operating parameters are not optimized, the jet rebound can be witnessed^44,45^, which we have avoided by optimizing the jetting parameters. After achieving the successful drop deposition, the effect of gravity on droplet spreading and equilibrium shape, in particular, for droplets with larger volume are demonstrated by performing a comparative study. The theoretical model is based on overall energy balance equation and can successfully predict the spreading dynamics while the droplet is forming via jet based deposition system. The detailed derivation and discussion on the model are presented in the Supplementary Discussion section.

### Dynamics of drop spreading

**Fig. 2:**
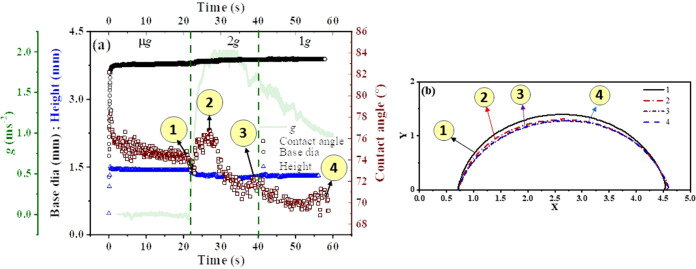
Transient variation of droplet shape and wetting properties with respect to different levels of gravity. Panel (**a**) is the representation of change in drop base diameter, drop height and dynamic contact angle with respect to time at micro-g (μ*g*), 2*g* and 1*g*, while (**b**) is the representation of change in drop profile, extracted from experimental still images, corresponds to the point 1, 2, 3 and 4 as shown in (**a**).

In this flight campaign (funded through FAST) we deposited drops of different volumes that distinguish the role of gravity on drop spreading and equilibrated drop shape configurations. Figure 2 represents the effect of gravity on the evolution of a 10 μl sessile droplet, generated by the liquid needle dosing system. This figure represents the state of a drop, generated by liquid needle drop deposition technique, in three different gravity levels, provided during the parabolic flight. As shown in Fig. 2, the microgravity environment stays from 0 to 22 s. It is to be noted that the drop deposition was initiated, as soon as the microgravity condition started, which was identified by the operator’s experience of gravity in relation to the announcements made by the pilot. For the 10 μl drop, the opening of the nozzle was closed at 660 ms, which ceased the jetting subsequently. Until this moment, the continuous influx of flow through the drop-medium interface, normal to the advancement of the three-phase contact line results in a rapid increasing in the drop base diameter and height. At the onset of the deposition (Supplementary Video 1), the drop grows vertically faster than the spreading along the substrate, hence, the contact angle increase at first for a few milliseconds and then decreases. This can be attributed to the slower rate of spreading at beginning which can be due to several reasons, such as; contact angle hysteresis, roughness of the substrates, jetting parameters, etc. Once the spreading begins, the base diameter and the maximum height of the drop grow until the volume is added to the drop. After the deposition of a required volume, the drop remained stable during the microgravity period but the marginal influence of g-jitter can be witnessed in contact angle variations.

The geometrical profile of the sessile drop on the copper substrate, while it is going through the μg, ~2*g*, and 1*g* is sequentially numbered in Fig. 2a whereas the corresponding extracted drop shape is shown in Fig. 2b. As a standard operating procedure followed by pilots for such parabolic flights, immediately after the microgravity, the flight goes through a 45^∘^ nose down manoeuvre, which results in ~2*g*. This ~2*g* or hypergravity period continued from 22 to 40 s. From 23.5 to 27 s, during this period, the drop base was pinned, however, due to the hypergravity the drop gets compressed and as a result an increase in contact angle is observed. In the event of drop pinning the base diameter remains unchanged, however, the height of the drop and subsequently contact angle will change. When the pinning effect is dominant, the drop profile deviates from the spherical cap and resembles an oblate ellipsoidal cap. From the experimental results presented in Fig. 2a, we can observe that during the *μg* period, both base diameter and height attain equilibrium at the same instant, one can argue that pinning was minimal. However, in hypergravity (~2*g*), when drop height decreases the base diameter does not increase proportionally, which suggests the pinning of the droplet. For an ideal substrate, the change in the base diameter with a constant contact angle can be witnessed. The contact angle attains another maxima (~78^∘^) at point 2 as annotated in Fig. 2a, and the corresponding profile of the drop at point 2 can be observed in Fig. 2b. After 27s the base diameter of the drop was increasing, while the height of the drop was decreasing and as a result the contact angle decrement continues until 40 s, as indicated by case 3 in Fig. 2a, b. Point 3 also marks the end of hypergravity period. After the hypergravity period the flight entered into the 1g period and during 1g period the contact angle remains stable at 71^∘^.

The hydrostatic pressure at the apex and base of a sessile drop is minimum and maximum, respectively. The variation from zero to maximum is a linear function of drop height measured from the apex of the drop^46^. For a given substrate-liquid combination, the hydrostatic pressure increases as the drop volume increases. At the three-phase contact line, the hydrostatic (*P*_H_) and capillary pressure (*P*_C_) act against each other. The hydrostatic pressure pushes the drop outward, whereas the capillary pressure attempts to minimize the surface area of the drop. With terrestrial gravity or hyper-gravity, drop with Bo > 1, the hydrostatic pressure is larger than capillary pressure and the difference grows as the droplet volume increases. During 2*g* manoeuvre, for larger droplets, the role of hydrostatic pressure is prominent. This can be witnessed by observing the decrease in drop height or bulging out of the droplet. However, a similar argument is invalid in μ*g*. This phenomenon is also well supported by our experimental observation in Fig. 2a. It is evident that under 2*g* drop height is smaller and in microgravity, it increases, which is the manifestation of pure surface tension-driven phenomenon.

During the hypergravity or 2*g* period, the drop get compressed from apex to the depth of the base due to the higher hydrostatic pressure. As a result the base or spreading diameter increases. From Fig. 2a we can also observe that during hypergravity, from 22 to 23.5 s, a sudden jump in base dia is observed, which is the manifestation of hydrostatic pressure-driven phenomena, because during this hypergravity period, the hydrostatic pressure is greater than the capillary pressure and as a result we can observe an outward flow along the base diameter.

**Fig. 3:**
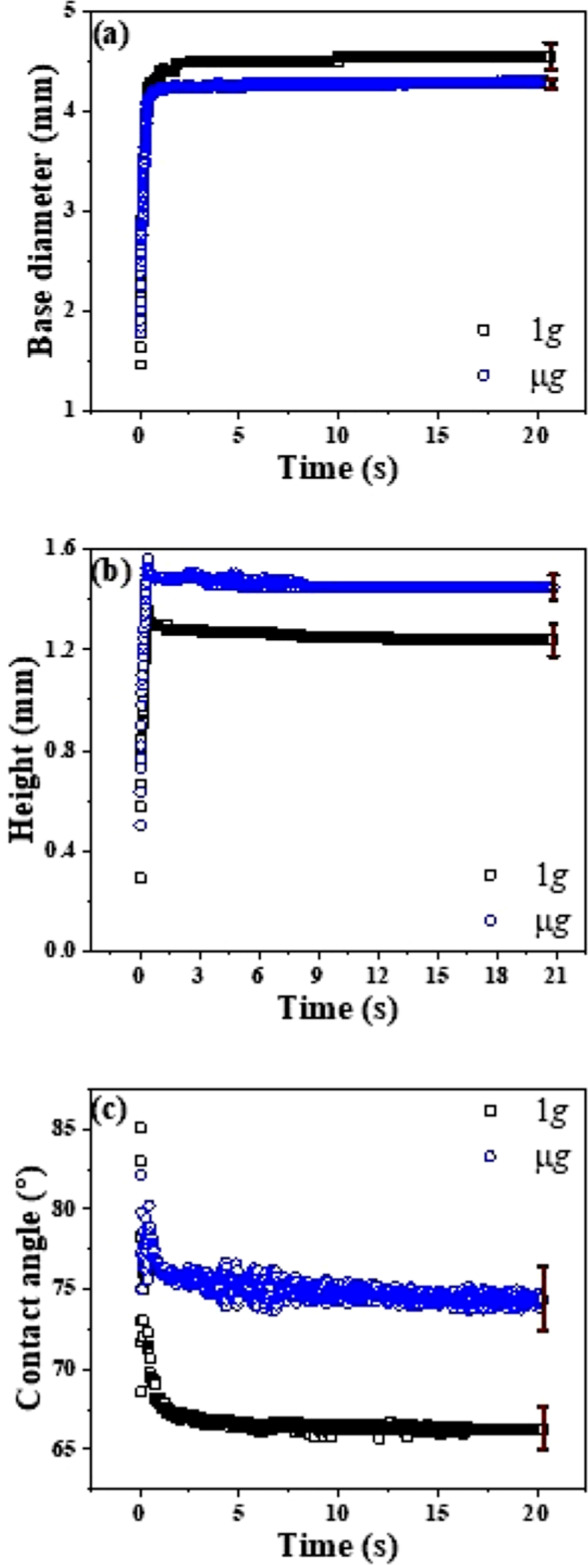
Variation of geometrical parameter of drop in *μg* and 1*g*. Comparison of (**a**) drop diameter (**b**) drop height and (**c**) contact angle with respect to time between μ*g* and 1*g*. The error bar presented in the figure imply the highest error in the corresponding dataset.

In order to distinguish the drop behaviour in microgravity from terrestrial gravity, we have presented Fig. 3. This figure compares the transient variation of physical parameters of the droplet for both the gravity conditions. From Fig. 3a, a slight difference in the transient variation of base diameter between microgravity (μg) and terrestrial gravity (1*g*) condition is observed. Under terrestrial gravity condition, the base diameter is larger than the micro gravity condition while the droplet is forming. It is noteworthy to mention that, the perimeter of the droplet where the three phases: liquid, solid and vapour meet, also called three phase contact line (TPCL), can be quantified based on the droplet base diameter if we make a well-accepted assumption of spherical cap shape. Under microgravity condition the surface tension dominates over the gravity and as a result the droplet tends to form a spherical shape and will minimize the solid-liquid and liquid-air surface area. Thus, in reduced gravity environment for a given drop volume, particularly for the drops having characteristic length larger than capillary length scale, the base diameter or TPCL spreads less as opposed to spreading with earth gravity as shown in Fig. 2. One can also argue that that contact angle hysteresis more prone in reduce gravity that restricts the drop spreading in pronounced way.

Figure 3b shows the variation of height with respect to time under *μ*g as well as 1*g*. A 10 μl volume of water droplet was formed on the copper substrate, by the continuous addition of mass in the form of water jet. From Fig. 2b, it is evident that the overall height of the droplet is significantly higher in the microgravity condition compared to the height in the earth gravity. While the droplet is in microgravity condition, the effects driven by capillary forces are enhanced due to which the droplet tends to stretch along its height rather spreading along the three-phase contact line by overcoming the pinning due to the contact angle hysteresis, which is measured as 38^∘^, for the tested substrate. Therefore, the droplet minimizes its base radius and tries to form a sphere over the substrate due to the interfacial forces. In the drop formation process (Supplementary Video 1), the drop under microgravity tends to grow normal to the three-phase contact line; therefore, the height of the drop is higher in the microgravity conditions. Under microgravity conditions, the drop is more stable than the terrestrial drop deposition, which is evident by the monotonous change in the height vs time plot, as well as it can also be observed that after ~9 s the drop stops oscillating in the vertical direction. On the other hand, the change in the drop height is not monotonous in terrestrial gravity. In 1*g*, after ~1 s, the drop height was reducing and it became stable after ~13 s. It indicates that under the effect of gravitational field, for drop volume as high as 10 μl, the competition between gravity and surface forces allow the drop reaching equilibrium later than the microgravity condition.

The influence of gravity on the advancing contact angle can also be understood from Fig. 3c. The significant difference of 8^∘^ in the contact angle can be easily observed from this figure. During the microgravity, the advancing contact angle is 74. 5^∘^ ± 2^∘^, whereas under terrestrial gravity the value of advancing contact angle is 66^∘^ ± 1. 3^∘^. The reason behind the higher contact angle, for a specific liquid-substrate combination, can also be explained with the help of Fig. 3b, c. Due to the absence of the gravitational effect, the pure surface tension-driven phenomenon takes place and as a result the deposited drop in microgravity tends to form a spherical bead to stay at a low energy state by contracting its base diameter and expanding its height. Therefore, a higher value of contact angle is observed in the microgravity condition. In contrast, inside the gravitational field, the hydrostatic force of the drop is no longer negligible rather for a larger drop it dominates over surface forces and the effect of hydrostatic force causes the drop to spread more and further reduces the contact angle. It has already been demonstrated that due to hydrostatic force the advancing contact angle decreases with the larger drop size, where Bond number is close to unity^47^.

In any ideal experimental condition contact angle depends on the measurement conditions, such as drop size, external forces (e.g. gravity)^47^, drop deposition rate^39^, characteristics of the solid needle material^28^, surface tilt angle^48^. In short, it can be said in ideal case, an experimental conditions in which drop based study is conducted does not guarantee that thermodynamic equilibrium is really achieved and as a result one can argue that it is not possible to measure advancing contact angle experimentally. Therefore, in order to distinguish between theoretical and experimentally achieved contact angle, apparent as placed contact angle is introduced^47^. As placed contact angle refers to a contact angle that a drop makes upon being placed gently on a horizontal surface, and after allowing some time for the drop to equilibrate, and pin to the surface in some metastable position somewhere between theoretical advancing and receding contact angle^47^. Therefore, contact angle resulted from the gravitational effect can be considered as an ‘as placed’ contact angle. It has also been observed that as placed contact angle is lower in magnitude than the true advancing contact angle. The results presented in Fig. 3c is representing a 10 μl drop. However, similar kind of results can also be shown for other volumes, as shown in Supplementary Fig. 2.

Again referring to Fig. 2 we can observe that in the parabolic flight experiment when the drop is in the microgravity period it sustains its low energy states by smaller base radius and larger drop height resulting in a higher contact angle (~74^∘^), whereas when the same drop enters into the gravity period the contact angle reduces to ~68^∘^, which is closer to the results observed in Fig. 3.

Again, the size and shape of the liquid droplet sticking to a solid substrate relies on the contact line hysteresis, pinning, and the stress balance at the two interfaces of the droplet, primarily the solid-liquid interface or at the three-phase contact line. The balance of the liquid droplet over a surface is affected by the gravity effect, surface tension and bulk flow inside the liquid drop. With gravity-effect together with surface tension, i.e., Bond number, induce the bulk flow, which could eventually influence the contact line and free surface shape of the liquid drop.

It is important to note that, under microgravity condition, simulated in drop tower or parabolic flight, the experimental set-up continuously going through vibrations, due to which it experiences a periodic time dependant acceleration, which is also called ‘g-jitter’. Due to the g-jitter or vibration of the plane body, the drop-medium also oscillates randomly. Therefore, in microgravity the experimental reproducibility and corresponding error are higher that on earth observation as observed in most of the literature related to droplet dynamics in reduced gravity environment^30,31,49^.

Considering the thorough investigation of the spreading dynamics of a droplet on a solid surface under the effect of terrestrial gravity and microgravity condition, performed in this study, the liquid needle droplet deposition technique is proved to be an ideal drop deposition technique in microgravity. From the experimental investigation, larger droplet height is evident for the microgravity compared to the terrestrial gravity condition. The theoretical model, which has accounted for the viscous force, the surface forces, the wettability of the substrate, and the gravitational force, successfully predicts the transient variation of the base diameter of the droplet. The study presented here can be considered as a potential element for answering some unanswered questions, especially in the field of jetting or material deposition through jetting for reduced gravity applications. With enough resources, it will be possible to extend the study to droplet coalescence and droplet manipulation in reduced gravity condition.

## Methods

### Experimental constraints and requirements

The choice of equipment used in devising the prototype experimental set-up was a result of constraints and requirements set by the National Research Council of Canada (NRC) in association with Canadian Space Agency (CSA). Size of the prototype was restricted to the dimensions of 45.7 × 45.7 × 45.7 cm^3^ to fit within a commercial protective case (Pelican Case Model 0340, Pelican Products Inc.), modified for parabolic flight, and weight of the prototype was 45 kg without the hard case. Power consumption was restricted to 600 W while power supply was provided in 115 VAC 5 Amp format. Batteries used in this experiment were restricted to the dry cell type. The same set-up was used for the ground-based experiment. To maintain the same operating conditions from temperature and humidity perspective, special arrangements and efforts were made such as installing the 18*L* (~5 Gallon) of desiccants for 45.7 × 45.7 × 45.7 cm^3^ pelican case which ascertain the relative humidity of 4–8%. Finally, during the parabolic flight, the cabin was pressurized to maintain the atmospheric pressure which is similar to the pressure that we have for the ground based experiment. Further, a major constraint was 20−23 s (typical) microgravity time. Considering the parabolic trajectory of the aircraft (Supplementary Fig. 1), constraints due to physical limitations caused procedures to be limited during ~2*g* manoeuvres, as well as the duration of the flight dictates the endurance limits of the experimenters. A detailed description of parabolic flight trajectory can be found in Supplementary Note 1.

### Prototype components

**Fig. 4:**
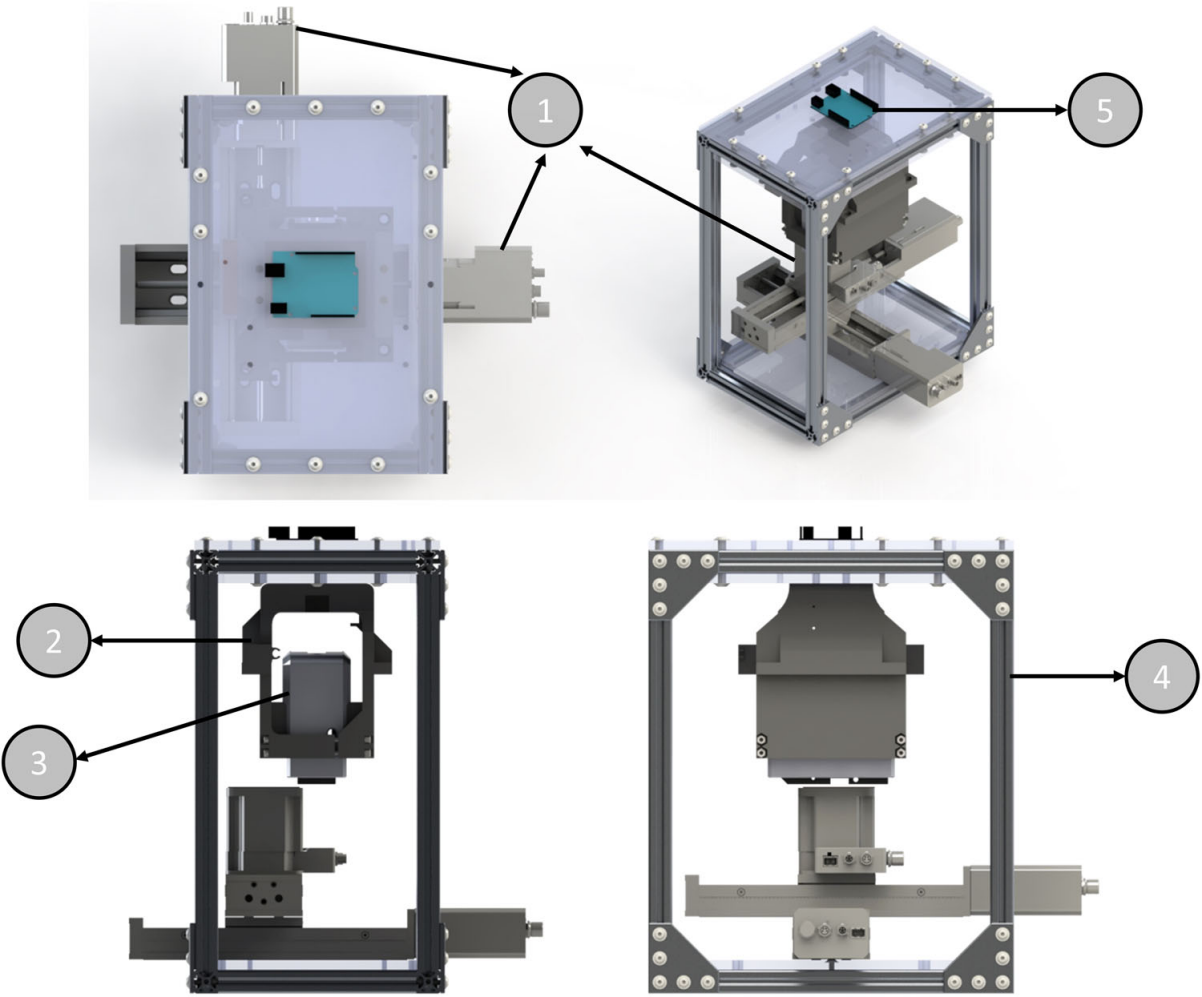
Experimental setup. Breakdown of key components: 1—motorized XYZ stage (QTY 2 X-LSQ150A and QTY 1 X-VSR20A, Zaber Technologies Inc.), 2—custom 3D printed MSA holder, 3—pressure dosing system (Mobile Surface Analyzer (MSA), KRÜSS Scientific Instruments, Inc.), 4—frame with vibration resistant frame mounts (not shown), 5—accelerometer.

The experimental set-up was assembled using components chosen as a result of the aforementioned constraints and requirements. During the event of parabolic trajectory the prototype must be able to deposit a drop, record video of the phenomenon at a frame rate suitable to capture all events, move the substrate to a new position to allow for multiple data points, record the acceleration experienced to confirm the events occurred in micro-gravity and control the humidity during the experiment. An overview of the experiment inside the hard case can be found in Fig. 4.

The main component of the experimental setup is commercial-off-the-shelf (COTS) the liquid jetting unit, Mobile Surface Analyzer (MSA, KRÜSS Scientific Instruments Inc.). The MSA was modified to accommodate a high-speed camera (UI-3060CP Rev. 2, IDS Imaging Development Systems GmbH). The upgraded camera allows events to be captured at 166 frames per second (fps) at 1936 × 1216 (full frame) and up to 2000 fps at 96 × 64. For the experiment, 400 frames per second were captured at 800 × 200 (cropped frame).

In this experiment, a commercial electromagnetic, 2/2-way modular microvalve, normally closed has been used as a dispensing nozzle. The microvalve is primarily actuated via electromagnetic force and the internal diameter of the nozzle is 0.1 mm. This nozzle is integral component of the commercially used pressure dosing system or mobile surface analyser (MSA, KRÜSS Scientific Instruments Inc.) and the necessary operating parameters, for efficient functioning of this nozzle, are controlled through the KRÜSS ADVANCE software^39^.

Further COTS components include a pair of linear motion stages with built-in controllers (QTY 2 X-LSQ150A, Zaber Technologies Inc.) couple with additional linear motion stage for *Z*-axis (QTY 1 X-VSR20A, Zaber Technologies Inc.). The *XYZ* stages reposition substrate between each parabola. To allow the MSA to dispense droplets without contacting the previously formed droplets, a 25 mm section of the MSA base was milled to provide clearance. The data acquisition system (DAQ) system monitors the environment for the period of reduced gravity and gives an indication to the operator when the drop should be deposited by using the monitoring command prompt. Accelerations in the *X*, *Y* and *Z* directions as well as quaternion rotations (later converted into Yaw, Pitch and Roll Euler angles) and angular rates (in degree per seconds (dps)) were recorded at 100 Hz throughout the flight and stored in a text-based log-file. Comparing the timestamps of each series of data allows us to verify that the events of interest have occurred in the reduced gravity environment. As mentioned earlier, to control the humidity, desiccant packs were loaded into the hard case to absorb the moisture and reduce humidity. A copper (Mirror-Like Multipurpose 110 Copper Sheet, P/N: 9821K31, McMaster Carr) substrates was firmly fixed to the Z axis using a commercially available thermal paste (Arctic Silver 5, silver compound thermal paste). Copper substrate was functionalized using a flaming process, which allowed the removal of organic contaminations. The stability of identical substrates and respected surface energy over period of time was verified using polar and non-polar liquids (details are provided in Supplementary Methods).

### Experimental procedure

The procedure followed to conduct the experiment is divided into 3 phases: Pre-flight, In-flight, and post-flight.

#### Pre-flight

In preparation for the flight, desiccants were placed in the hard case for minimum of 24 h before the flight occurred. A measurement of the humidity within the hard case was taken and was found to be in the range of 4–8% in the morning before the flight. Prior to take-off, the software was loaded and the functionality of the devices was tested.

#### In-flight

Before the first experimental parabola took place, the log file for the accelerometer was started. Operating the prototype required a strict procedure to be followed due to the sequential g-forces (Supplementary Note 1 and Supplementary Fig. 1). During the 2*g* periods, motor function of the operators diminishes; to avoid strain on the operator and to avoid potential operator error, procedural steps were restricted to the level flight and the microgravity periods. Following the parabola of the Falcon 20 parabolic flight, the in-flight procedure planned as follows. During the level flight and before initial 2*g* pull (pre-microgravity), operator queues camera recording software and waits for the 2*g* pull indication then starts camera recording. Drop deposition triggers when microgravity is reached. Directly after remaining 2*g* pull (post-microgravity), operator stops recording, indexes the drop deposition unit to the next position and changes drop volumes if necessary. This procedure is repeated for each parabola until the end of the flight.

#### Post-flight

Once the last parabola has been completed, the log files were stopped, duplicates of the data were stored on an external memory device, and the devices were disconnected from the laptop. The data was then processed later to obtain the results of interest. To process the data, various methods were used from using rudimentary spreadsheet analysis for using sophisticated software to analyse the data. The data from the log files required simple arithmetic operations to produce the data. In this study, we have used two commercial image processing software to measure the geometrical parameter of the droplet, which includes contact angle, height, base diameter and volume. In order to measure the contact angle, axisymmetric drop shape analysis (ADSA) system has been adopted. ADVANCE (KRÜSS Scientific Instruments Inc.) software has been used to perform the contact angle angle measurement by adopting tangent droplet method. On the other hand, we used ImagePro (media cybernetics), to quantify the drop radius and height. The outer diameter of the nozzle is considered as a reference for the calibration.

### Experimental Trails

The crucial factor for the success of this experiment was the deposition of a droplet onto a surface under microgravity conditions. Without this functionality, the experimental set-up would not have been able to produce the anticipated results. Concerns that the droplet may not detach from the dosing orifice existed, but the pressure dosing unit within the MSA was capable of ejecting the droplet. The supply pressure of the MSA created a large enough force for the liquid to properly detach from the dosing unit without any unwarranted effects like the formation of the satellite droplets or rebounding of the jet. With the known mass flow rate, jet diameter (10 μm) and measured volume of the droplet we can determine the jet velocity. It is evident that in both the cases, the required drop growth is achieved at the same time for a constant drop volume. This assures us that the jet speed is not significantly altered due to the gravitational effects.

**Fig. 5:**
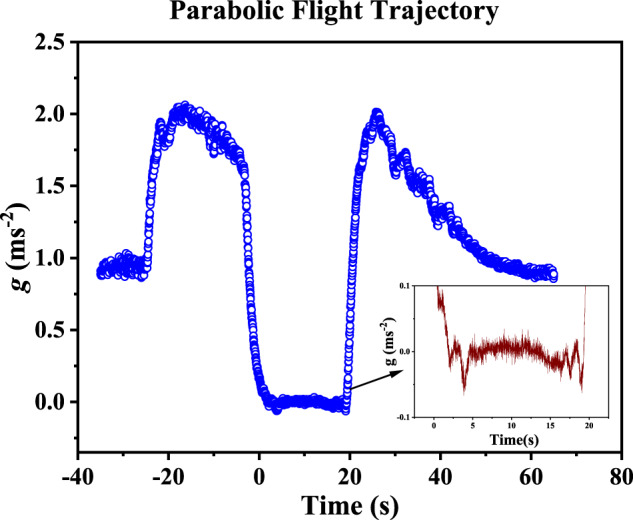
Gravitational acceleration profile in parabolic flight. Gravitational acceleration as a function of time at vertical *Z*-axis recorded during flight. In comparison to *Z*-axis acceleration along *X* and *Y* axes are negligible and; therefore, we have omitted them. The inset figure represents the gravitational and g-jitter value during the parabolic path of the flight or in microgravity environment.

With the Falcon 20, operated for parabolic manoeuvre, 8 parabolas were planned to perform the planned experiments. Figure 5 represents the acceleration versus time plot at different coordinates and it is evident that the we attained the magnitude of gravity as low as 2 × 10^−5^*g*. From the inset figure we can also get the information about the strength of the periodic time dependant acceleration i.e., g-jitter, the maximum value of which is ± 0.07*g*. For the better prediction from the propose model, this g-jitter profile was used as gravitational acceleration for the theoretical modelling.

### Reporting summary

Further information on research design is available in the Nature Research Reporting Summary linked to this article.

## Mathematical model

As mentioned earlier, the mechanism of droplet generation via liquid needle is free from solid-needle and droplet interaction.Now considering all of the forces involved in jetting process, we can develop a theoretical model for droplet spreading, based on overall energy balance (OEB) approach as suggested by Erickson et al.^50^. By adopting OEB approach the dimensional form of the governing equation that dictates the spreading of the droplet, deposited by liquid needle drop deposition technique, under gravity can be expressed by Eq. (1), the detailed derivation of which can be found in the Supplementary Discussion section.

1 $$\begin{array}{l}6\pi {\mu }_{{{{\rm{d}}}}}\ln \left({\varepsilon }^{-1}\right)\frac{R}{{\theta }_{{{{\rm{d}}}}}}{\left(\frac{{\rm{d}}R}{{\rm{d}}t}\right)}^{2}\\ +\frac{{\rm{d}}m}{{\rm{d}}t}\left[\frac{gRf({\theta }_{{{{\rm{d}}}}})}{4}-\frac{{v}_{{{{\rm{j}}}}}^{2}}{2}\right]\\ +\left[2\pi R{\sigma }_{{{{\rm{dm}}}}}(2h({\theta }_{{{{\rm{d}}}}})-\cos {\theta }_{{{{\rm{e}}}}})+\left({m}_{0}+\frac{{\rm{d}}m}{{\rm{d}}t}\right)g\frac{f({\theta }_{{{{\rm{d}}}}})}{4}-\frac{4{\mu }_{{{{\rm{d}}}}}}{R{\rho }_{{{{\rm{m}}}}}}\frac{{\rm{d}}m}{{\rm{d}}t}\right]\frac{{\rm{d}}R}{{\rm{d}}t}=0\end{array}$$

In equation (1), *μ*_d_ and *μ*_m_ is the viscosity of the droplet and surrounding medium, respectively and *ε* is the ratio of the microscopic length (*L*_*δ*_) to macroscopic cut-off length (*L*)^50^. In general, *L*_*δ*_ may vary between 1 *μ*m to 5 *μ*m, whereas *L* can be defined as the characteristic length scale (*R*) of the drop. The advancing (dynamic) and equilibrium contact angle of the droplet can be denoted by *θ*_d_ and *θ*_e_, respectively, and $f({\theta }_{{{{\rm{d}}}}})=\frac{2-{\sin }^{2}{\theta }_{{{{\rm{d}}}}}\,+\,2\cos {\theta }_{{{{\rm{d}}}}}}{(2\,+\,\cos {\theta }_{{{{\rm{d}}}}})\sin {\theta }_{{{{\rm{d}}}}}}$, whereas, *σ*_*dm*_ is the interfacial tension between drop-medium interface, *m* is the total mass of the deposited droplet at any time *t*, *ρ*_m_ is the mass density of the droplet, *D*_j_ is the diameter of the jet and *v*_j_ is the velocity of the impacting jet.

The non-dimensional form of equation (1) can also be obtained as depicted in equation (2) where the characteristic length and velocity are considered as the jet radius and velocity, respectively.

2 $$\begin{array}{ll}\frac{6\ln ({\varepsilon }^{-1})}{{\theta }_{{{{\rm{d}}}}}}\frac{{R}^{* }}{{{{\rm{Re}}}}}{\left(\frac{{\rm{d}}{R}^{* }}{{\rm{d}}{t}^{* }}\right)}^{2}\\ +\left[\frac{4{R}^{* }}{{{{\rm{We}}}}}(2h({\theta }_{{{{\rm{d}}}}})-\cos {\theta }_{{{{\rm{e}}}}})\right.\\ +\frac{f({\theta }_{{{{\rm{d}}}}})G({\theta }_{{{{\rm{d}}}}})}{24}{({R}_{0}^{* })}^{3}\frac{{{{\rm{Bo}}}}}{{{{\rm{We}}}}}+\frac{{k}_{{h}_{{{{\rm{j}}}}}}f({\theta }_{{{{\rm{d}}}}})}{4}{t}^{* }\frac{{{{\rm{Bo}}}}}{{{{\rm{We}}}}}\\ \left.+\frac{{k}_{{\mu }_{{{{\rm{m}}}}}}}{{R}^{* }}\frac{8}{{{{\rm{Re}}}}}\right]\frac{{\rm{d}}{R}^{* }}{{\rm{d}}{t}^{* }}+\frac{{\rm{d}}m}{{\rm{d}}t}\left[\frac{f({\theta }_{{{{\rm{d}}}}})}{4}{R}^{* }\frac{{{{\rm{Bo}}}}}{{{{\rm{We}}}}}\right]=0\end{array}$$

Here, Reynolds number $({{{\rm{Re}}}})$ is *ρ*_d_*v*_j_*D*_j_/*μ*_d_, Weber number $({{{\rm{We}}}})$ is ${\rho }_{{{{\rm{d}}}}}{v}_{{{{\rm{j}}}}}^{2}{D}_{{{{\rm{d}}}}}/{\sigma }_{{{{\rm{dm}}}}}$, and Bond number $({{{\rm{Bo}}}})$ is ${\rho }_{{{{\rm{d}}}}}g{D}_{{{{\rm{j}}}}}^{2}/{\sigma }_{{{{\rm{dm}}}}}$; also, viscosity ratio $({k}_{{\mu }_{{{{\rm{m}}}}}})$ is *μ*_m_/*μ*_d_. Further details of non-dimensional parameters such as ${R}^{* },{R}_{0}^{* }$, and t^*^ can be found in the Supplementary Discussion.

It is to be noted that the first term in both Eqs. (1) and (2) represent the viscous dissipation work, which is based on lubrication approximation theory^51^. However, lubrication approximation applies when equilibrium contact angle is less than 90^∘^. Therefore, for higher contact angle scenario, the boundary layer approximation^52^ should be used. For boundary layer approximation the first term in both equations should be replaced by ${\mu }_{{{{\rm{d}}}}}{v}_{{{{\rm{j}}}}}\pi {k}_{{h}_{{{{\rm{j}}}}}}\sqrt{{{{\rm{Re}}}}}R\frac{{\rm{d}}R}{{\rm{d}}t}$ and $\frac{{k}_{{h}_{{{{\rm{j}}}}}}}{4\sqrt{{{{\rm{Re}}}}}}{R}^{* }\frac{{\rm{d}}{R}^{* }}{{\rm{d}}{t}^{* }}$, respectively, where, ${k}_{{h}_{{{{\rm{j}}}}}}={h}_{{{{\rm{j}}}}}/{D}_{{{{\rm{j}}}}}$.

As the governing equation either (1) or (2) is a non-linear ordinary differential equation, the numerical solution is strongly dependent on the initial condition of the system. We have considered the maximum drop diameter of the droplet, *R*_0_, at the first onset of the impact on the substrate, i.e., the splat shape of the drop as the initial condition. With the knowledge of droplet impact analysis, the splat shape of the drop can be non-dimensionalised and can be expressed as initial spreading ratio, *ξ* = *D*_0_/*D*_j_ = *R*_0_/*R*_j_. In the case of an impacting jet, we can assume that at the first instant, the splat-shape drop spreading is obtained with a drop volume equivalent to initial jet volume immediately before impact from the nozzle to the surface. The non-dimensional equation of initial spreading ratio (*ξ* = *D*_0_/*D*_j_) can be calculated for both lubrication and boundary layer approximation, from the energy balance equation, as shown in Eqs. (3) and (4), respectively. The detailed derivation of initial spreading ratio (*ξ* = *D*_0_/*D*_j_), in case of jet-based deposition technique can be found in the Supplementary Discussion.

3 $${\xi }^{3}\left[\frac{9ln({\varepsilon }^{-1})}{32{\theta }_{{{{\rm{d}}}}}}+\frac{3{k}_{{\mu }_{{{{\rm{m}}}}}}{k}_{{h}_{j}}}{8}\right]\frac{{{{\rm{We}}}}}{{{{\rm{Re}}}}}+\frac{{\xi }^{2}}{4}[1-\cos {\theta }_{{{{\rm{e}}}}}]-\frac{{k}_{{h}_{{{{\rm{j}}}}}}}{8}{{{\rm{We}}}}-{k}_{{h}_{{{{\rm{j}}}}}}-\frac{1}{4}{k}_{{h}_{{{{\rm{j}}}}}}^{2}{{{\rm{Bo}}}}=0$$

4 $$\frac{1}{4}\frac{{k}_{{\mu }_{{{{\rm{m}}}}}}}{{k}_{{h}_{{{{\rm{j}}}}}}}\frac{{{{\rm{We}}}}}{{{{\rm{Re}}}}}{\xi }^{3}+\left[\frac{{{{\rm{We}}}}}{8\sqrt{{{{\rm{Re}}}}}}+\frac{1}{4}(1-{\rm{cos}}({\theta }_{{{{\rm{e}}}}}))\right]{\xi }^{2}-\frac{{k}_{{h}_{{{{\rm{j}}}}}}}{8}{{{\rm{We}}}}-\frac{1}{4}{k}_{{h}_{{{{\rm{j}}}}}}^{2}{{{\rm{Bo}}}}=0$$

**Fig. 6:**
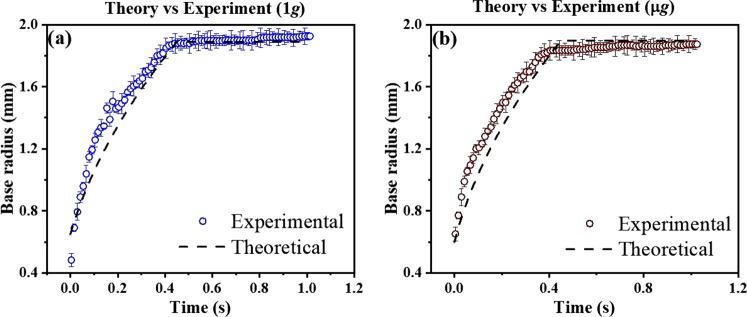
Validation of theoretical model with experimental observation. Comparison between theoretical prediction and experimental observation of droplet spreading under (**a**) terrestrial gravity and (**b**) reduced gravity environment.

Figure 6 compares the theoretical prediction presented in this paper with the experimental observations. From this figure, we can say that considering the error bar our theoretical model can successfully predict the effect of gravity on the spreading of the droplet. Taking a closer look at Fig. 6, it can be observed that our theoretical model slightly under predict the drop spreading in microgravity, the reason for which can be attributed to the fact that in the model for the sake of simplicity the effect of g-jitter was ignored.

It is noteworthy to mention that one of the major assumptions in our theoretical model is that we assume the drop profile will be a spherical cap. However, the spherical cap assumption depends on the Bond number. The spherical cap assumption is invalid if the Bond number, Bo > 1. When the Bo is above unity a transition from a spherical cap to the paddle shape is observed for a sessile drop^53^. From the perturbation solution approach it has been observed that the sessile drop profile starts deviating from the spherical cap once the corresponding length of the Bond number is 0.8 × *l*_cap_^54^. The deviation becomes pronounced once the Bo is corresponding to the 2.4 × *l*_cap_, at which the drop becomes paddle or splat shape. From the sensitivity analysis it can be shown that the theoretical model presented in this study can predict the physics of drop dynamics until Bo ≈ 2.

## Supplementary information

Supplementary Video

Supplementary Information

Reporting Summary

## Data Availability

The data that support the findings of this study are available from the corresponding author upon reasonable request.

## Code availability

The code (Matlab ODE45) used to generate the theoretical plots is available from the authors upon reasonable request.
