# Supplementary material for: Effect of gravity on the spreading of a droplet deposited by liquid needle deposition technique
Source: NPJ Microgravity. 2023 Jun 21;9:49. doi: 10.1038/s41526-023-00283-2 (PMC10284902; doi:10.1038/s41526-023-00283-2)
Supplement: Supplementary file 2 — Supplementary Information [file 41526_2023_283_MOESM2_ESM.pdf]

Supplementary Materials

Supplementary Discussion

This supplementary section represents the detailed derivation of the governing equation and the associated boundary conditions, presented in the main text. In case of liquid needle droplet deposition technique the energy imparted by the impinging jet is transformed into internal energy, surface energy, and gravitational energy, in addition to the resistance offered by the medium viscosity and viscous dissipation within the spreading droplet. The energy transferred from the impinging jet to the spreading drop results in equation 1 as follows:

$$\frac{dE_{\text{in}}}{dt} = \frac{d}{dt}(E_{\text{system}} + E_{\text{s}} + E_g) + \frac{d}{dt}(W_{\text{vd}} + W_{\text{mv}}) \tag{1}$$

Where, E_{in}, E_{system}, E_{s} and E_g is the incoming energy available in the liquid jet, the internal energy in the spreading droplet, the surface energy and the gravitational potential energy, respectively, whereas, W_{vd} and W_{mv} is the work due to viscous dissipation and the work associated with medium viscosity, respectively.

It is to be noted that the kinetic energy of the jet is the only incoming energy, E_{in} to the system. Considering the mass of the incoming liquid as m and velocity of the impacting jet as, v_{j}, the rate of change of incoming energy transfer can be expressed as,

$$\frac{dE_{\text{in}}}{dt} = \frac{v_{\text{j}}^2}{2}\frac{dm}{dt} \tag{2}$$

The internal energy of the spreading droplet is the combination of enthalpy [1] and the kinetic energy of the jet. If we assume the overall system as isobaric and isothermal system, then from order of magnitude analysis it can be demonstrated that the overall change in the enthalpy is negligible [2]. on the other hand, the internal kinetic energy induced due to the

impingement of the jet on the liquid-medium interface can be ignored since the drop surface area is remarkably greater than the liquid jet cross sectional area. Therefore, as both the enthalpy and internal kinetic energy are negligible, the internal energy in the system can be considered as constant. As a result, in equation 1 the transient change in E_{system} can be ignored.

The total surface energy of the system can be defined considering the surface energies of the three interfacial phases, *i.e.*, liquid-solid (drop-substrate), liquid-fluid (drop-medium) and solid-fluid (substrate-medium) which suggests $E_s = \sigma_{\text{ds}} A_{\text{ds}} - \sigma_{\text{sm}} A_{\text{sm}} + \sigma_{\text{dm}} A_{\text{dm}}$, where, σ and A represent the surface energy and area for respective interfaces and subscripts d, s and m denotes the drop, solid and the surrounding medium, respectively. With a spherical drop shape assumption the rate of change of surface energy can be expressed as,

$$\frac{dE_{\text{s}}}{dt} = 2\pi R \sigma_{\text{dm}} [2h(\theta_{\text{d}}) - \cos\theta_{\text{e}}] \frac{dR}{dt} \quad (3)$$

where, $h(\theta_{\text{d}}) = \dfrac{1 - \cos\theta_{\text{d}}}{\sin^2\theta_{\text{d}}}$ and θ_{d} and θ_{e} are the advancing (dynamic) and equilibrium contact angle, respectively.

During droplet growth, due to the continuous addition of mass, the change in the mass and the change in the center of gravity of the depositing droplet the consideration of the gravitational forces become mandatory. For an infinitesimal increase of mass, Δm, if the center of gravity is shifted by Δz, the change in gravitational potential energy can be expressed as, $\Delta E_g = g[(m + \Delta m)(z + \Delta z) - mz]$. After eliminating the negligible products ($\Delta m.\Delta z \approx 0$) the rate of change in potential energy can be expressed as, $\dfrac{dE_g}{dt} = g\left[m\dfrac{dz}{dt} + z\dfrac{dm}{dt}\right]$,where the total mass of the system can be obtained as $m = m_0 + \int_a^b \dfrac{dm}{dt}dt$. Now considering all the terms the expression for the rate of change in the gravitational potential energy can be

expressed as,

$$\frac{dE_g}{dt} = g\left[m\left(\frac{3f(\theta_\mathrm{d})}{4}\frac{dR}{dt}\right) + \frac{R}{4}f(\theta_\mathrm{d})\frac{dm}{dt}\right] \tag{4}$$

where, $f(\theta_\mathrm{d}) = \dfrac{2 - \sin^2\theta_\mathrm{d} + 2\cos\theta_\mathrm{d}}{(2+\cos\theta_\mathrm{d})\sin\theta_d}$.

The viscosity of the surrounding medium can play a crucial role in confining the spreading of the droplet. During liquid needle droplet deposition, due to the continuous addition of mass, the surrounding medium get displaced by the increase in drop volume. Therefore, additional work has been done by the surrounding medium on the drop-medium interface. The rate of work due to medium viscosity can be quantified as, $\dfrac{dw_\mathrm{mv}}{dt} = \tau V dA$, where τ is the shear stress which acts normal to the surface, dA, due to the drop spreading velocity, $V = dR/dt$. The shear stress at the drop boundary, i.e. at the liquid-medium interface, can be defined as $\tau = 2\mu_\mathrm{m}\left(\dfrac{\partial u}{\partial r}\right)_{r=R}$, where, μ_m is the medium viscosity and u is the velocity by which the surrounding medium is getting displaced. If we consider a lamina of fluid outside the drop at a distance r, where $r > R$, and implement the mass conservation between surrounding medium and the droplet, one can obtain the velocity of the surrounding medium adjacent to the drop boundary as, $u = \dfrac{R^2}{r^2}\dfrac{dR}{dt}$. The resultant rate of work due to medium viscosity can be represented as,

$$\frac{dw_\mathrm{mv}}{dt} = \left[\frac{4\mu_\mathrm{m}}{R}\frac{dR}{dt}\frac{1}{\rho_\mathrm{m}}\right]\frac{dm}{dt} \tag{5}$$

If we are to consider the internal motion of the liquid layers inside a droplet, the consideration of viscous dissipation force is inevitable. Depending on the magnitude of the contact angle, two models have been adopted by the researchers: lubrication approximation [3, 4] or boundary layer approximation [5]. De Gennes predicted the viscous dissipation work based on lubrication approximation. For hydrodynamic drop spreading when the contact angle of

the droplet is less than 90°, we follow the De Gennes approach. Based on the lubrication model [3,4], the viscous force per unit length of the three-phase contact line can be expressed as, $F_{\mathrm{v}} = \frac{3\mu_{\mathrm{d}}}{\theta_{\mathrm{d}}} ln\left(\varepsilon^{-1}\frac{dR}{dt}\right)$, where θ_{d} is the instantaneous dynamic contact angle, μ_{d} is the viscosity of the droplet and ε is the ratio of the microscopic length (L_δ) to macroscopic cut-off length (L). In general, L_δ may vary between 1 μm to 5 μm whereas L can be defined as the horizontal length scale (R) of the drop [2]. The viscous dissipation work of the circular three phase contact line is $2\pi R F_{\mathrm{v}}$. Therefore, the rate of viscous dissipation work over the three-phase contact line can be expressed as [3,4],

$$\frac{dw_{\mathrm{vd}}}{dt} = 6\pi\mu_{\mathrm{d}} ln(\varepsilon^{-1})\frac{R}{\theta_{\mathrm{d}}}\left(\frac{dR}{dt}\right)^2 \tag{6}$$

On the other hand, as an alternative to lubrication approximation, boundary layer approach can be taken into consideration for predicting the viscous dissipation for drop-substrate combination of higher contact angle. Boundary layer approximation for viscous dissipation model was suggested by Chandra et al. [5]. Based on the boundary layer approximation model the viscous dissipation work is approximated as, $w_{\mathrm{v}} = \int_a^b \phi\Omega t_{\mathrm{c}}$. [5–7], where ϕ is the viscous dissipation which can be approximated as,$\phi = \mu_{\mathrm{d}}\left(\frac{\partial v_{\mathrm{i}}}{\partial x_{\mathrm{k}}} + \frac{\partial v_{\mathrm{k}}}{\partial x_{\mathrm{i}}}\right)\frac{\partial v_{\mathrm{i}}}{\partial x_{\mathrm{k}}} = \mu_{\mathrm{d}} v_{\mathrm{j}}^2/\delta^2$, where $\delta = \frac{2D_{\mathrm{j}}}{\sqrt{Re}}$ is the characteristic length scale of the droplet, $\Omega = \pi R^2\delta$ is the volume of the droplet and $t_{\mathrm{c}} = h_{\mathrm{j}}/v_{\mathrm{j}} = k_{h_{\mathrm{j}}} D_{\mathrm{j}}/v_{\mathrm{j}}$. Therefore, considering the boundary layer approximation, the work done due to viscous dissipation per unit time, over the three-phase contact line, can be expressed as,

$$\frac{dw_{\mathrm{vd}}}{dt} = \mu_{\mathrm{d}} v_{\mathrm{j}} \pi k_{h_{\mathrm{j}}} \sqrt{Re} R \frac{dR}{dt} \tag{7}$$

Now, considering the lubrication approximation model for the maximum wetting scenario, combining equation 1 to 6, we can form the following governing equation for the drop spreading for liquid needle drop deposition technique,

$$6\pi\mu_{\mathrm{d}} ln(\varepsilon^{-1})\frac{R}{\theta_{\mathrm{d}}}(\frac{dR}{dt})^2 + [2\pi R\sigma_{\mathrm{dm}}(2h(\theta_{\mathrm{d}}) - \cos\theta_{\mathrm{e}})$$
$$+(m_0 + \frac{dm}{dt})g\frac{f(\theta_{\mathrm{d}})}{4} - \frac{4\mu_{\mathrm{m}}}{R\rho_{\mathrm{m}}}\frac{dm}{dt}]\frac{dR}{dt}$$
$$+\frac{dm}{dt}[\frac{gRf(\theta_{\mathrm{d}})}{4} - \frac{v_{\mathrm{j}}^2}{2}] = 0 \quad (8)$$

Whereas, considering the boundary layer approximation theory in case of the minimum wetting scenario, combining equation 1 to 5 and 7, we can form the following governing equation for the drop spreading for liquid needle drop deposition technique,

$$\mu_{\mathrm{d}} v_{\mathrm{j}}\pi k_{h_{\mathrm{j}}}\sqrt{Re}R\frac{dR}{dt} + \Bigg[2\pi R\sigma_{\mathrm{dm}}(2h(\theta_{\mathrm{d}}) - \cos\theta_{\mathrm{e}})$$
$$+(m_0 + \frac{dm}{dt})g\frac{f(\theta_{\mathrm{d}})}{4} - \frac{4\mu_{\mathrm{m}}}{R\rho_{\mathrm{m}}}\frac{dm}{dt}\Bigg]\frac{dR}{dt}$$
$$+\left[\frac{gRf(\theta_{\mathrm{d}})}{4} - \frac{v_{\mathrm{j}}^2}{2}\frac{dm}{dt}\right] = 0 \quad (9)$$

The non-dimensional form of governing equation can be expressed as the following equation 10 and 11, for lubrication and boundary layer approximation, respectively.

$$\frac{6ln(\varepsilon^{-1})}{\theta_{\mathrm{d}}}\frac{R^*}{Re}\left(\frac{dR^*}{dt^*}\right)^2 + \left[\frac{4R^*}{We}(2h(\theta_{\mathrm{d}}) - \cos\theta_{\mathrm{e}}) + \frac{f(\theta_{\mathrm{d}})G(\theta_{\mathrm{d}})}{24}(R_0^*)^3\frac{Bo}{We}\right.$$
$$\left. + \frac{k_{h_{\mathrm{j}}} f(\theta_{\mathrm{d}})}{4}t^*\frac{Bo}{We} + \frac{k_{\mu_{\mathrm{m}}}}{R^*}\frac{8}{Re}\right]\frac{dR^*}{dt^*} + \frac{dm}{dt}\left[\frac{f(\theta_{\mathrm{d}})}{4}R^*\frac{Bo}{We}\right] = 0 \quad (10)$$

$$\frac{k_{h_{\mathrm{j}}}}{4\sqrt{Re}}R^*\frac{dR^*}{dt^*} + \left[\frac{4R^*}{We}(2h(\theta_{\mathrm{d}}) - \cos\theta_{\mathrm{e}}) + \frac{f(\theta_{\mathrm{d}})G(\theta_{\mathrm{d}})}{24}(R_0^*)^3\frac{Bo}{We} + \frac{k_{h_{\mathrm{j}}} f(\theta_{\mathrm{d}})}{4}t^*\frac{Bo}{We}\right.$$
$$\left. + \frac{k_{\mu_{\mathrm{m}}}}{R^*}\frac{8}{Re}\right]\frac{dR^*}{dt^*} + \frac{dm}{dt}\left[\frac{f(\theta_{\mathrm{d}})}{4}R^*\frac{Bo}{We}\right] = 0 \quad (11)$$

Where, $Re = \rho_{\mathrm{d}} v_{\mathrm{j}} D_{\mathrm{j}}/\mu_{\mathrm{d}}$, $We = \rho_{\mathrm{d}} v_{\mathrm{j}}^2 D_{\mathrm{j}}/\sigma_{\mathrm{dm}}$, and $Bo = \rho_{\mathrm{d}} g D_{\mathrm{j}}^2/\sigma_{\mathrm{dm}}$ are Reynolds number, Weber number and Bond number, respectively; also, $k_{\mu_{\mathrm{m}}} = \mu_{\mathrm{m}}/\mu_{\mathrm{d}}$, $R^* = \dfrac{R}{D_{\mathrm{j}}/2}$, $R_0^* = \dfrac{R_0}{D_{\mathrm{j}}/2}$, $t^* = \dfrac{t}{D_{\mathrm{j}}/v_{\mathrm{j}}}$ and $G(\theta_{\mathrm{d}}) = \dfrac{2 - 3\cos(\theta_{\mathrm{d}}) + \cos^3(\theta_{\mathrm{d}})}{\sin^3(\theta_{\mathrm{d}})}$.

The spreading of the droplet or the transient variation of the droplet base radius can be predicted by numerically solving (e.g. 4th order Runge Kutta (RK4)method) either equation 10 or 11, with corresponding contact angle value and appropriate initial boundary condition. For this study we have chosen the initial condition as the drop radius at the moment when the jet makes the first impact on the substrate, i.e., the splat shape of the droplet. Here, we assume that the volume of the splat shape droplet is equivalent to the volume of the liquid jet before the impact.

To determine the initial spreading diameter of the splat, we can further employ the energy balance equation, *i.e.* the energy available in the jet (kinetic energy) before deposition and the energy transferred (surface energy, viscous dissipation work and work done due to medium viscosity) to form the splat droplet shape (initial drop shape). The kinetic energy of the impacting jet can be calculated as, $\dfrac{1}{2} m v_{\mathrm{j}}^2$. We can define the surface energy considering the splat shape as, $\frac{\pi}{4} D_0^2 \sigma_{\mathrm{dm}}(1 - \cos\theta_{\mathrm{e}})$. Considering De Genne's approximation [3,4] the viscous dissipation work can be calculated as, $6\pi\mu_{\mathrm{d}} ln(\varepsilon^{-1}) \dfrac{R}{\theta_{\mathrm{d}}} \left(\dfrac{dR}{dt}\right)^2$. Here, we can approximate the change in radius and time as $dR \approx D_0/2$ and $dt \approx t_{\mathrm{c}}$. The time required for the droplet to form splat shape (t_{c}) can be readily used from the traditional droplet impact analysis [8]. Finally, the work due to medium viscosity considering the elemental area of the splat can be approximated as $\dfrac{3\pi k_{\mu_{\mathrm{m}}}}{8} \dfrac{k_{h_{\mathrm{j}}} D_0^3 We}{D_{\mathrm{j}} Re}$.
Thus, the non-dimensional equation for the initial spreading ratio ($\xi = D_0/D_{\mathrm{j}}$) is:

$$\xi^3 \left[\frac{9ln(\varepsilon^{-1})}{32\theta_{\mathrm{d}}} + \frac{3k_{\mu_{\mathrm{m}}} k_{h_{\mathrm{j}}}}{8}\right] \frac{We}{Re} + \frac{\xi^2}{4}[1 - \cos\theta_{\mathrm{e}}] - \frac{k_{h_{\mathrm{j}}}}{8} We - k_{h_{\mathrm{j}}} = 0 \tag{12}$$

Again, by replacing only the viscous dissipation work, approximated by lubrication model, with the boundary layer approximation, we can write the non-dimensional energy balance equation for the splat formation as,

$$\frac{1}{4}\frac{k_{\mu_{\rm m}}}{k_{h_{\rm j}}}\frac{We}{Re}\xi^3 + \left[\frac{We}{8\sqrt{Re}} + \frac{1}{4}(1 - cos(\theta_{\rm e}))\right]\xi^2 - \frac{k_{h_{\rm j}}}{8}We = 0 \tag{13}$$

Supplementary Note 1

Parabolic flights generate gravity free conditions in an aircraft by following a parabolic trajectory as shown in supplementary figure 1 . They provide a microgravity environment for scientists to conduct research without reaching the outer space but only for 20-25 seconds. During a parabolic or zero gravity flight the aircraft makes a parabolic manoeuvre to achieve a state of weightlessness for minimum 10 seconds to maximum 22 seconds. The parabolic manoeuvre or ellipse arc during a zero gravity flight is divided into three stages: the parabola pull-up, the parabola, and the parabola pull-out as shown in figure.

Before performing a parabolic manoeuvre, the aircraft needs to be at the $6000m$ while it is in the horizontal flight mode as well as the aircraft needs to gradually gain a speed of $800 - 900\ kmh^{-1}$ before the pull up stage.

During the pull up stage, the pilot lifts the nose of the aeroplane upward from its horizontal position to an angle of 45°. The pull up stage lasts for 20 seconds while the whole aircraft system experiencing a pull of 1.8 times that of gravity on Earth.

As the aircraft travel upwards during the pull up stage, the pilot gradually reducing the speed from $800 - 900\ kmh^{-1}$ to $685\ kmh^{-1}$. Approximately at an altitude of $7800m$ the aircraft enters the parabolic trajectory, as shown in figure, during which it is in free fall for 22 seconds.

Substrate Type	W-CA [°]	DIIM-CA [°]	SFE $[mNm^{-1}]$	Polar part $[mNm^{-1}]$	Dispersive part $[mNm^{-1}]$
CUCL	91.61	69.29	26.51	3.24	23.27
CUCLF	33.42	31.15	69.02	25.27	43.74
CUCLF-W1	48.42	35.73	59.81	18.15	41.66
CUCLF-W16	59.91	35.74	53.46	11.17	41.69

Table 1: Table depicting the water contact angle (W-CA), diiodomethane contact angle (DIIM-CA), surface free energy (SFE), polar and dispersive part of variously treated copper substrate.

During the pull out stage the nose of the plane is tilted back to downward to 45° and during this period the pilot gradually start increasing the flight speed until the plane level off. Whole system in the aeroplane again experience a pull of 1.8 times that of gravity on Earth.

Supplementary Methods

Copper substrates used in the study underwent routine testing for historical changes in surface free energy (SFE) using a portable goniometer (Mobile Surface Analyzer (MSA), Krüss Scientific Instruments Inc.) equipped with polar and nonpolar liquids, such as water and diiodomethane (DIIM). As shown in the Table 1 CUCL (Copper cleaned) is a copper substrate cleaned with isopropanol and distilled water. CUCLF(Copper cleaned flamed) undergoes the same treatment with the addition of a flaming step at the end of the process. W1 and W16 stand for week number 1 and 16, respectively, since a substrate has been treated with the flame.

It can be seen from the results that a significant increase in both polar and dispersive parts can be achieved using the flaming process. The dispersive part exhibits insignificant change over sixteen (16) weeks unlike the polar part that reduced by nearly fifty-five (55) percent.

Supplementary Figures

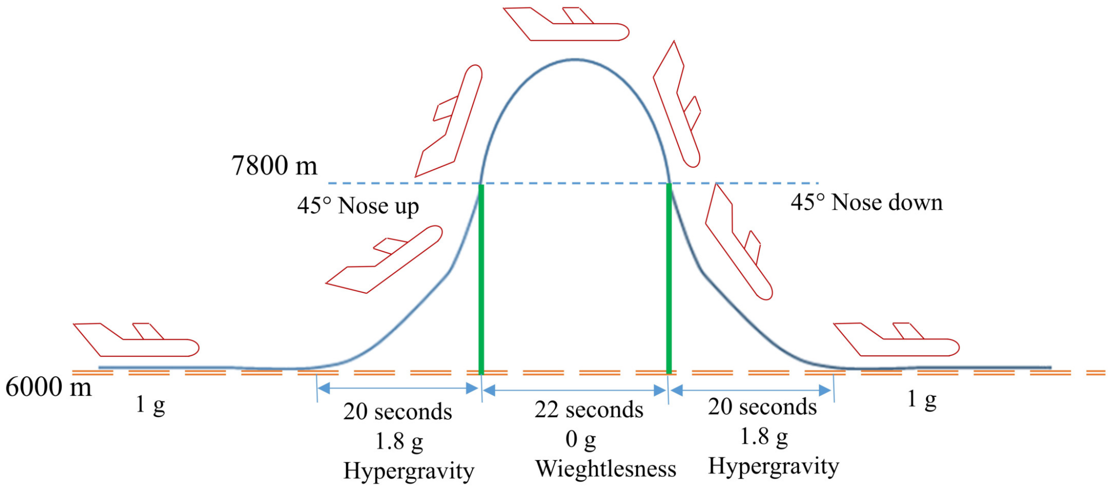

Supplementary Figure 1: **Parabolic flight trajectory.** Parabolic flight trajectory and the period of hyper-gravity and weightlessness.

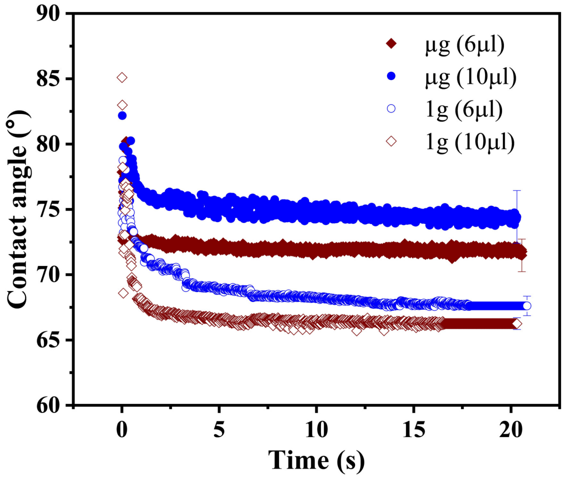

Supplementary Figure 2: **Contact angle as a function of volume in different gravitational level.** Variation of dynamic contact angles for different volumes as well as in different gravity level.

References

[1] Sonntag, R. E., Borgnakke, C., Van Wylen, G. J. & Van Wyk, S. Fundamentals of thermodynamics, vol. 6 (Wiley New York, 1998).

[2] Erickson, D., Blackmore, B. & Li, D. An energy balance approach to modeling the hydrodynamically driven spreading of a liquid drop. Colloids Surf. A Physicochem. Eng. Asp. **182**, 109–122 (2001).

[3] De Gennes, P.-G. Wetting: statics and dynamics. RMP **57**, 827 (1985).

[4] Brochard-Wyart, F. & De Gennes, P. Dynamics of partial wetting. Adv. Colloid Interface Sci. **39**, 1–11 (1992).

[5] Chandra, S. & Avedisian, C. On the collision of a droplet with a solid surface. Proc. R. Soc. Lond. A Math. Phys. Sci. **432**, 13–41 (1991).

[6] Jin, M., Sanedrin, R., Frese, D., Scheithauer, C. & Willers, T. Replacing the solid needle by a liquid one when measuring static and advancing contact angles. Colloid Polym. Sci. **294**, 657–665 (2016).

[7] Ahmed, A., Fleck, B. A. & Waghmare, P. R. Maximum spreading of a ferrofluid droplet under the effect of magnetic field. Phys. Fluids **30**, 077102 (2018).

[8] Pasandideh-Fard, M., Qiao, Y., Chandra, S. & Mostaghimi, J. Capillary effects during droplet impact on a solid surface. Physics of fluids **8**, 650–659 (1996).
